# Guidance for the evaluation and treatment of hereditary and acquired thrombophilia

**DOI:** 10.1007/s11239-015-1316-1

**Published:** 2016-01-16

**Authors:** Scott M. Stevens, Scott C. Woller, Kenneth A. Bauer, Raj Kasthuri, Mary Cushman, Michael Streiff, Wendy Lim, James D. Douketis

**Affiliations:** Department of Medicine, Intermountain Medical Center, 5121 Cottonwood Street, Murray, UT 84157-7000 USA; Department of Internal Medicine, University of Utah, Salt Lake City, UT USA; Beth Israel Deaconess Medical Center, Harvard Medical School, Boston, MA USA; Johns Hopkins Comprehensive Hemophilia Treatment Center, Baltimore, MD USA; Department of Medicine, Cardiovascular Research Institute of Vermont, University of Vermont, Burlington, VT USA; Department of Medicine, University of North Carolina at Chapel Hill, Chapel Hill, NC USA; Department of Medicine, McMaster University, Hamilton, ON Canada

**Keywords:** Thrombophilia, Hereditary thrombophilia, Antiphospholipid syndrome, Venous thromboembolism, Risk factors

## Abstract

Thrombophilias are hereditary and/or acquired conditions that predispose patients to thrombosis. Testing for thrombophilia is commonly performed in patients with venous thrombosis and their relatives; however such testing usually does not provide information that impacts management and may result in harm. This manuscript, initiated by the Anticoagulation Forum, provides clinical guidance for thrombophilia testing in five clinical situations: following 1) provoked venous thromboembolism, 2) unprovoked venous thromboembolism; 3) in relatives of patients with thrombosis, 4) in female relatives of patients with thrombosis considering estrogen use; and 5) in female relatives of patients with thrombosis who are considering pregnancy. Additionally, guidance is provided regarding the timing of thrombophilia testing. The role of thrombophilia testing in arterial thrombosis and for evaluation of recurrent pregnancy loss is not addressed. Statements are based on existing guidelines and consensus expert opinion where guidelines are lacking. We recommend that thrombophilia testing not be performed in most situations. When performed, it should be used in a highly selective manner, and only in circumstances where the information obtained will influence a decision important to the patient, and outweigh the potential risks of testing. Testing should not be performed during acute thrombosis or during the initial (3-month) period of anticoagulation.

## Introduction

Thrombophilias are hereditary or acquired conditions which can increase the risk of venous or arterial thrombosis. As the etiology of thrombosis is multifactorial, the presence of a thrombophilic defect is only one of many elements that determine risk. Therefore, the utility of testing for thrombophilia to inform prevention and treatment decisions is controversial. In this guidance document, we will review evidence and provide guidance regarding thrombophilia testing to inform clinical decisions regarding duration of anticoagulation following venous thromboembolism (VTE) and primary prevention of VTE in relatives of affected patients. Testing for thrombophilia in the context of arterial thrombosis, and recurrent pregnancy loss is not addressed. As no randomized, prospective trials have tested the utility of testing for thrombophilia, evidence is taken from epidemiologic studies.

## Background

### Hereditary thrombophilias

The term “hereditary” or “inherited” thrombophilia has most commonly been applied to conditions in which a genetic mutation affects the amount or function of a protein in the coagulation system. Loss of function mutations include those affecting antithrombin (AT), protein C (PC) and protein S (PS) [1–3]. Gain of function mutations include the factor V Leiden (FVL) and the prothrombin gene 20210 A/G (PGM) mutations [4, 5]. Testing for these defects will be addressed in this guidance statement. We will not address other conditions, such as abnormal levels of other coagulation proteins (e.g., elevated factor VIII levels) elevated homocysteine, or abnormalities of fibrinolytic proteins. Over time it is likely that more defects will be identified [6]. This guidance statement also does not address hereditary factors not assessed in the laboratory, such as gender, height, leg length, and body mass index; which also affect thrombosis risk [7–10].

### Acquired thrombophilias

The antiphospholipid syndrome (APS) is the sine qua non example of an acquired thrombophilia, and guidance statements address testing for this condition. While not specifically addressed herein, many other acquired conditions can increase the risk of thrombosis, such as acquired abnormalities in coagulation proteins (e.g. deficiencies in the natural anticoagulants, activated protein C resistance in the absence of FVL) and certain diseases (e.g. myeloproliferative neoplasms, paroxysmal nocturnal hemoglobinuria, cancer). Some medications increase thrombosis risk, including exogenous hormones and chemotherapy [11]. Acquired characteristics such as smoking, obesity, increasing age and pregnancy also increase risk [11].

### Strong vs. weak thrombophilias

Some authors have classified thrombophilias as “strong” or “weak” based on the magnitude of thrombosis risk conferred. It should be noted that published VTE guidelines differ in these classifications [12–14] and that uncertainty exists because of the broad confidence intervals around risk estimates for the more rare thrombophilias [15–18]. The risk of thrombosis conferred by a given thrombophilia may vary by circumstance (e.g. in pregnancy) [19–24]. Depending on the classification system, it may not be possible to identify instances of strong thrombophilia without also identifying the more common weak cases. For example, based on the high prevalence of FVL, as many as 128 heterozygotes (considered a weak thrombophilia) would be found for every homozygote.

### Thrombophilia testing

Testing for thrombophilias should only be performed when results will be used to improve or modify management. Testing has been suggested to assist with secondary prevention (determining the duration of anticoagulation following a thrombotic event); and for hereditary disorders, to aid in primary prevention in relatives of affected patients. Guidance statements herein are based on analysis of the utility and disutility of positive or negative test results to inform these decisions. Disutility primarily results from a decision to withhold anticoagulants from patients at high risk for thrombosis (placing them at risk of a thrombotic event); or providing anticoagulants to patients at low risk of thrombosis (placing them at risk for bleeding). Other potential types of disutility include cost, spurious results due to inappropriate timing of testing [25], misinterpretation of results (by patients or healthcare providers) [26–28], emotional distress and anxiety [28, 29], and the possibility of genetic discrimination [30]. Potential utilities include patient satisfaction from having identification of a biologic risk factor underlying a thrombotic event, and an increased likelihood of using prophylaxis in high risk situations by affected relatives [31, 32]. The presence of hereditary thrombophilia does not affect survival in patients with a history of VTE [33] or the risk of post-thrombotic syndrome [34].

## Methods

We chose to address five questions relevant to thrombophilia testing to inform clinical decisions regarding primary and secondary prevention for VTE, and one question regarding the timing of testing. A literature search of MEDLINE from January 1990 to July 2015 was conducted, restricted to publications in English. The following search terms were used and combined: lupus anticoagulant, antiphospholipid, antithrombin, protein S, protein C, prothrombin gene mutation, activated protein C resistance, prothrombin G20210A, factor V Leiden, thrombophilia, pulmonary embolism, venous thrombosis, deep vein thrombosis, venous thromboembolism, primary prevention, secondary prevention. When meta-analyses, reviews or guideline articles were identified, the reference lists of these were reviewed for additional citations of interest. We preferred prospective cohort studies to inform guidance statements when available, but guidance could also derive from case–control and retrospective studies. Table 1 summarizes the guidance questions in this chapter.

**Table 1:** Guidance questions to be considered

Secondary prevention following provoked VTE
Should thrombophilia testing be performed to help determine duration of anticoagulation following provoked VTE?
Secondary prevention following unprovoked VTE
Should thrombophilia testing be performed to help determine duration of anticoagulation following unprovoked VTE?
Primary prevention in relatives of VTE patients
Should family members of patients with VTE or hereditary thrombophilia undergo thrombophilia testing?
Primary prevention in female relatives of VTE patients considering estrogen
Should female relatives of patients with VTE or hereditary thrombophilia who are considering using estrogen-containing medications be tested for thrombophilia?
Primary prevention in female relatives of VTE patients who are contemplating pregnancy
Should female relatives of patients with VTE or hereditary thrombophilia who are contemplating pregnancy be tested for thrombophilia?
Timing of thrombophilia assessment
When thrombophilia testing is performed, at what point in the patient’s care should this be done?

## Guidance

*Secondary prevention following provoked VTE*

1. Should thrombophilia testing be performed to help determine duration of anticoagulation following provoked VTE?

Of the many factors which predict the risk of recurrent thrombosis after an initial event, the presence of provoking factors is the most important [12, 20]. Thrombosis following a major provocation, such as major surgery in the preceding 3 months, carries a short term relative risk of recurrence of 0.5 or less compared to the absence of an identifiable provocation [20]. A large prospective registry offered patients with VTE the opportunity to be screened for hereditary thrombophilia. The risk of recurrence following an episode of provoked VTE was very low, and recurrence rates did not differ among those with or without a hereditary thrombophilia [35]. A large case–control study and another prospective registry yielded similar results [16, 22]. Therefore, the risk:benefit balance favoring time-limited anticoagulation remains the same, regardless of the presence of a thrombophilia. There is the potential for patient harm if thrombophilia testing is performed after a provoked VTE event, as healthcare providers may overestimate the risk of recurrence and prescribe extended duration anticoagulation, subjecting patients to the unnecessary risk of bleeding [36, 37]. Other published guidelines broadly agree that thrombophilia testing does not assist with clinical decision making in cases of provoked VTE and should not be performed [21, 25, 38, 39]. The American Society of Hematology (ASH) and the Society for Vascular Medicine (SVM) recommended against testing in such cases in their Choosing Wisely© initiatives [36, 37].

### **Guidance Statement**

*Do not perform thrombophilia testing following an episode of provoked VTE.*

*Remark*

A positive thrombophilia evaluation is not a sufficient basis to offer extended anticoagulation following an episode of provoked VTE.

*Secondary prevention following unprovoked VTE*

(2) Should thrombophilia testing be performed to help determine duration of anticoagulation following unprovoked VTE?

The absolute risk for recurrent VTE among patients with unprovoked thrombosis is higher than among those with provoked VTE, with 5-year risk approaching 30 % unless extended-duration anticoagulant therapy is provided [20, 40]. Current guidelines from the American College of Chest Physicians (ACCP) recommend extended duration anticoagulation (anticoagulation with no planned stop date) after unprovoked VTE unless the risk of bleeding is high or this is contrary to the patient’s values and preferences [20]. However, anticoagulant medications confer an increased risk of major bleeding, inconvenience to patients, and not all patients will go on to develop recurrent thrombosis. Therefore it would be desirable to offer extended anticoagulation only to those who would benefit from it. Thrombophilia testing has been suggested as a means to identify these patients. However, a large registry did not demonstrate a difference in rates of recurrent VTE in patients who were tested for thrombophilia versus those who were not, although the number of patients with AT, PC, PS deficiencies or compound heterozygosity for FVL/PGM was limited [35]. Several prospective registries and case–control studies revealed clinically insignificant differences in the VTE recurrence rates for those with or without a hereditary thrombophilia [16, 22, 41, 42]. Some thrombophilias, including APS, confer a higher risk of recurrence than others (Table 2) and support the decision for extended anticoagulation. However, if a patient will remain on indefinite anticoagulation based on the known recurrence rate for unprovoked VTE, then thrombophilia testing may not add additional utility. Therefore, the value of testing is likely limited to patients who would stop anticoagulants unless they are at even higher risk of recurrence than the initial unprovoked event would predict. There are also potential risks to thrombophilia testing after unprovoked VTE. Negative testing for thrombophilia may falsely reassure clinicians that the risk of recurrent VTE is low after an unprovoked VTE, leading to cessation of anticoagulation in patients at high risk for recurrence [43]. Conversely, finding a thrombophilia in a patient at high bleeding risk may lead to continued anticoagulation, due to an overestimation of the risk conferred by the condition. Other published guidelines vary regarding thrombophilia testing after unprovoked VTE [15, 38]. The National Institute for Health and Clinical Excellence (NICE) guidelines discourage testing for FVL and PGM, and suggest selective testing for other conditions [21]. The ACCP guideline lists thrombophilias among factors which “…predict risk of recurrence, but not strongly or consistently enough to influence recommendations on duration of therapy” [20]. Several risk prediction models (which do not include thrombophilia testing) have been proposed to help inform decisions regarding duration of anticoagulation after unprovoked VTE; though some were derived in populations which intentionally excluded patients with known deficiency of PS, PC, AT and with APS [8, 44, 45]. As direct acting oral anticoagulants (DOACs) may confer a lower risk of bleeding than warfarin during extended therapy, the barriers to extended anticoagulation may be lessened, perhaps further decreasing the utility of thrombophilia status to inform clinical decisions. Other factors, such as the degree of post-thrombotic symptoms, D dimer levels after a minimum of 3 months of anticoagulant therapy, and residual vein thrombosis may also modify the risk of recurrence [46–48].

**Table 2:** Prevalence and thrombosis risk for selected thrombophilias

Thrombophilia	Prevalence	Relative (*absolute annualized*) risk of Initial VTE^a^	Relative risk of recurrent VTE	Relative (*absolute annualized*) risk of initial VTE, OCP users^a,b^	Relative (*absolute annualized*) risk of initial VTE, HRT users^a,b,c^	Relative (*absolute*) risk of initial VTE, pregnancy^a^
FVL Heterozygous	2–7 %	3.48–5.51 (0.05-0.2 %)	1.1–1.8	2.47–15.04 (0.1–0.6 %)	1.4–13.16 (1.6–5.97 %)	8.3 (0.8–4.6 %)
FVL Homozygous	0.06–0.25 %	6.79–19.29 (0.8 %)	1.8	Uncertain	Uncertain	34.4 (1.4–25.8 %)
PGM Heterozygous	1–2 %	2.25–3.48 (0.13 %)	0.7–2.3	3.60–8.63	(2.85 %)	6.8 (0.3–5.6 %)
PGM Homozygous	Rare	2.19–20.72	Uncertain	Uncertain	Uncertain	26 (0.2–78.3 %)
Compound FVL & PGM Heterozygosity	0.1 %	1.13–5.04 (0.42 %)	2.7	3.79–76.47 (0.17 %)	Uncertain	(4 %)
PC deficiency	0.2–0.5 %	10 (0.4–2.3 %)	1.8	1.7–23.9 (1.7–7.1 %)	(2.96 %)	4.8 (0.4–8.9 %)
PS deficiency	0.1–0.7 %	9.6 (0.7–3.2 %)	1.0	1.4–17.1 (1.3–2.4 %)	(2.3 %)	3.2 (0.2–14.7 %)
AT deficiency	0.02 %	10–30 (1.2–4.4 %)	2.6	1.4–115.8 (2.5–5.1 %)	(5.73 %)	4.7 (0.08–15.8 %)
APS	2 %	7	1.5–6.8	0.3–3.1	(1.05–2.63 %)	15.8

*OCP* oral contraceptive pill (containing estrogen), *HRT* hormone replacement therapy (containing estrogen), *VTE* venous thromboembolism, *FVL* factor V Leiden, *PGM* prothrombin Gene G20210A, *PC* protein C, *PS* protein S, *AT* antithrombin, *APS* antiphospholipid syndrome

^a^Data for are taken from several sources; absolute differences may therefore differ from calculations based on prevalence and relative risk [16, 17, 23, 32, 38, 50, 56, 62, 75–79]

^b^Relative risks are compared to non-users without thrombophilia

^c^With the exception of heterozygous FVL, estimates are based on modeling rather than epidemiologic studies

### **Guidance Statement**

*Do not perform thrombophilia testing in patients following an episode of unprovoked VTE.*

*Limits/exceptions*

If a patient with unprovoked VTE and low bleeding risk is planning to stop anticoagulation, test for thrombophilia (Table 2) if test results would change this decision.

*Remark*

A negative thrombophilia evaluation is not a sufficient basis to stop anticoagulants following an episode of unprovoked VTE in a patient with low bleeding risk and willingness to continue therapy.

*Remark*

Heterozygosity for FVL or PGM does not increase the predicted risk of recurrence after unprovoked VTE to a clinically significant degree.

See Chapter 3, “Guidance for the treatment of DVT and PE” for guidance on determining duration of anticoagulant therapy following unprovoked VTE.

*Primary prevention in relatives of VTE patients*

(3) Should family members of patients with VTE or hereditary thrombophilia undergo thrombophilia testing?

An additional rationale for hereditary thrombophilia testing of patients with VTE is to identify conditions which can lead to screening of asymptomatic family members. Except for temporary prophylaxis during certain high risk situations, anticoagulation for primary prevention of thrombosis is not advocated regardless of the genetic defect because the risk of bleeding may be higher than the absolute risk of a first thrombotic event [19, 49–51]. However, it is argued that people who know their genotype may be more likely to use preventive strategies in situations where the risk of thrombosis is elevated, such as during hospitalization, following major surgery, and during long distance travel. A large screening study of family members of VTE patients revealed that asymptomatic carriers of a hereditary thrombophilic defect were at excess risk of thrombosis, with risks varying by disorder [52]. However, a family history of thrombosis alone carries an increased risk, even in the absence of an identifiable thrombophilia [43, 53–55]. Therefore, negative thrombophilia screening does not equate to normal VTE risk. The impact of family screening on behavior was explored in a cohort of 382 first degree family members of patients with VTE and hereditary thrombophilia, who were tested and followed over about 9 years [32]. Twice as many thrombophilia carriers used prophylaxis in risk situations. The rate of provoked VTE was higher in the group with thrombophilia (0.58 %/year in those with hereditary thrombophilia; 0.24 %/year in those without) although this difference did not reach statistical significance (*p* = 0.08). This study suggested a potential harm from screening, as it demonstrated that family members who tested negative for a thrombophilic defect were less likely to use prophylaxis. Published guidelines vary substantially in regard to utility of family screening [21, 25, 38, 39].

### **Guidance Statement**

*Do not test for thrombophilia in asymptomatic family members of patients with VTE or hereditary thrombophilia.*

*Remark*

As a family history of VTE confers an excess risk of thrombosis, relatives should be counseled regarding use of prophylaxis in high risk situations.

*Primary prevention in female relatives of VTE patients considering estrogen*

(4) Should female relatives of patients with VTE or hereditary thrombophilia who are considering using estrogen-containing medications be tested for thrombophilia?

Thrombophilias act synergistically with the pro-thrombotic effects of estrogen-containing medications [19]. Therefore, identification of a hereditary thrombophilia by family testing could result in choice of an alternate method of contraception, or foregoing hormone replacement therapy (HRT), thus avoiding the associated risk of thrombosis. While the presence of thrombophilia significantly increases the relative risk of a thrombotic event during use of an estrogen-containing medication, the absolute risk remains low (Table 2). This is especially true for oral contraceptives (OCPs), which are generally used by younger women with a very low baseline risk for VTE. Studies focused on testing for thrombophilia in the general female population have suggested little utility and lack of cost-effectiveness. For example, an economic modeling study calculated that more 10,000 women would have to be screened for FVL, and 500 women with the condition would have to avoid OCPs in order to prevent one thrombotic event [56]. Over 92,000 FVL carriers would have to be identified and avoid OCPs to prevent one fatal pulmonary embolism (PE), at a cost of over $300 million [57]. Testing only patients with a family history would likely increase test yield and improve cost-effectiveness [56]. However, family history of VTE in one or more first degree relatives predicts an elevated risk of estrogen-associated VTE, regardless of whether thrombophilia is present [25, 53, 58]. Therefore it is possible that women with negative tests could be falsely reassured, and use estrogen in spite of an increased thrombosis risk. Other published guidelines vary significantly regarding family screening for the purposes of primary prevention in women contemplating estrogens. The NICE guidelines recommend against screening [21]. Medication package inserts contain precautions regarding estrogen-containing contraceptives in women with a family history of VTE. Intrauterine devices, including those which elute progestin, are a contraceptive option that does not increase the risk for thrombosis [31]. While HRT prescriptions have declined based on an unfavorable balance of risks and benefits [59], women with a family history of VTE who strongly desire HRT may mitigate VTE risk with use of a transdermal preparation [60].

### **Guidance Statement**

*Do not test for thrombophilia in asymptomatic family members of patients with VTE or hereditary thrombophilia who are contemplating use of estrogen.*

*Limits*/*exceptions*

If a woman contemplating estrogen use has a first-degree relative with VTE and a known hereditary thrombophilia (Table 2), test for that thrombophilia if the result would change the decision to use estrogen.

*Remark*

Family history of VTE in a first degree relative predicts an excess risk of thrombosis with estrogen use, even when thrombophilia testing is negative.

*Primary prevention in female relatives of VTE patients who are contemplating pregnancy*

(5) Should female relatives of patients with VTE or hereditary thrombophilia who are contemplating pregnancy be tested for thrombophilia?

Pregnancy is a period of particularly high risk for thrombosis, causing a relative risk increase of 5–10 times baseline [61]. The presence of a thrombophilic defect amplifies this risk several-fold further (Table 2). Thrombophilia screening, if performed, would be most applicable to the setting of primary prevention, as women with a prior VTE that was unprovoked, or provoked by pregnancy or an OCP, merit prophylaxis regardless of thrombophilia status [25, 62]. A personal history of a prior VTE provoked by surgery or trauma does not significantly increase the risk of VTE during pregnancy; and no special prophylaxis measures are indicated ante-partum [25, 62–64]. Pregnant patients with a first degree family member who has had VTE do not appear to have an excess risk of thrombosis in the absence of thrombophilia; therefore testing may be more likely to distinguish women at low or higher thrombosis risk [65, 66]. Screening of unselected pregnant women was not found to be cost-effective in a modeling analysis despite the assumption that all women who tested positive would use both antenatal and post-partum prophylaxis [19]. However, restricting testing to women with a first degree family member with VTE improved cost-effectiveness. A recent multinational prospective, randomized, open-label trial compared prophylaxis with dalteparin versus no prophylaxis in 289 pregnant women with thrombophilia who were at increased risk of placenta-mediated pregnancy complications, VTE, or both. Antepartum prophylactic dalteparin did not reduce the occurrence of VTE, pregnancy loss, or placenta-mediated pregnancy complications, but increased minor bleeding. All participants received post-partum prophylaxis with dalteparin [67]. Systematic reviews have concluded that the evidence supporting management decisions for pregnant patients with FVL or PGM is low [68], and that practitioners are often uncertain how to best manage these patients [27]. There are potential harms to testing. As prophylaxis is generally recommended only to women who harbor less common thrombophilias (see Chapter 6) [25, 62, 63], a large number of women must be screened to detect each case, resulting in significant expense [19]. Also, as homozygosity for FVL is one of the higher-risk conditions in pregnancy, screening will identify many heterozygotes in order to detect the few homozygotes of interest. The heterozygotes may experience worry, emotional distress or challenges with insurability [28–30], while gaining little or no utility from the information obtained. Other published guidelines vary in suggesting broad [15] or selective [25, 63] screening of relatives of patients with VTE who are contemplating pregnancy; and several guidelines advocate prophylaxis of pregnant women in the presence of certain thrombophilias [24, 62, 63, 69–71]; though the evidence underlying these recommendations has been questioned [68].

### **Guidance Statement**

*Do not test for thrombophilia in asymptomatic family members of patients with VTE or hereditary thrombophilia who are contemplating pregnancy.*

*Limits/exceptions*

If a woman contemplating pregnancy has a first-degree relative with VTE and a known hereditary thrombophilia (Table 2), test for that thrombophilia if the result would change VTE prophylaxis decisions.

*Remark*

Women with a personal history of unprovoked, estrogen-associated or pregnancy associated VTE already carry an indication for prophylaxis, and are unlikely to benefit from thrombophilia testing.

*Remark*

Women with multiple family members affected by VTE are more likely to carry a higher risk thrombophilia such as AT deficiency which may impact prophylaxis decisions.

See Chapter 6, “Guidance for the treatment of obstetric-associated VTE” for regimens recommended for prophylaxis based on history and thrombophilia status.

*Timing of thrombophilia assessment*

(6) When thrombophilia testing is performed, at what point in the patient’s care should this be done?

Genotype-based tests (such as those for FVL and PGM) and antibody titers (for cardiolipin and beta-2 glycoprotein I) can be performed accurately at any point in the care of a patient. Certain assays for lupus anticoagulants can be performed in the presence of heparins but others may return a false-positive result. A clinician contemplating this test should verify the assay used by the local laboratory before performing the test in the setting of therapy with heparin or low-molecular weight heparin. The remaining thrombophilia tests are influenced by the presence of acute thrombosis or anticoagulant therapy. Therefore, it is best to avoid testing for these thrombophilias in the setting of an acute VTE or while a patient is on an anticoagulant [31]. In the patient with VTE in whom thrombophilia testing has been chosen (see above), either deferring testing until anticoagulation has been stopped, or a two-stage approach is reasonable. In the two-stage approach, tests for thrombophilia that can be reliably done on anticoagulation (FVL, PGM, cardiolipin and beta-2 Glycoprotein-I antibodies) are performed before stopping anticoagulation. If these tests are normal, anticoagulation is discontinued and the remaining thrombophilia tests (lupus anticoagulant, PC, PS and AT) are performed. A final decision on disposition of anticoagulation can then be made on the basis of results. The time that anticoagulation must be interrupted before testing can take place is controversial, and may vary according to the anticoagulant being used [25, 26, 72]. One approach is to perform testing following a 2–4-week period off anticoagulation, which would match the common timing for D-dimer assessment if this is also being performed to assist in decision-making [46]. In the primary prevention setting, it is important to note that pregnancy strongly influences PS activity. It is unclear what PS activity value is diagnostic of deficiency in the pregnant patient, but thresholds have been suggested [24]. When testing is chosen (see above), testing prior to pregnancy is preferred [24]. Regardless of when tested, PS deficiency is difficult to diagnose, and clinicians should be familiar with the limitations of different assays, and consider seeking expert consultation to confirm this diagnosis [73]. It is important to note that the results of thrombophilia tests are frequently misinterpreted by physicians [26] so correct timing of testing and careful interpretation are essential [31, 74].

### **Guidance Statement**

1. *Do not perform thrombophilia testing at the time of VTE diagnosis or during the initial 3-month course of anticoagulant therapy.*
2. *When testing for thrombophilias following VTE, use either a 2-stage testing approach (see above) or perform testing after a minimum of 3 months of anticoagulant therapy has been completed, and anticoagulants have been held.*
  *Remark*
  *Pregnancy, sex and estrogen use reduce the levels of Protein S. Use of sex specific reference intervals, and testing prior to pregnancy or while not receiving estrogen preparations is preferred.*

## Conclusion

Thrombophilia testing is performed far more frequently than can be justified based on available evidence; the majority of such testing is not of benefit to the patient and may be harmful. Thrombophilia testing should not be performed in patients with VTE following a major provocation as extended anticoagulation is not indicated in these cases. Patients with unprovoked VTE are at sufficiently high risk of recurrent thrombosis that anticoagulation should be continued regardless of thrombophilia status, as long as bleeding risk is not high and treatment is consistent with the patient’s values and preferences. Testing may benefit select patients who would otherwise stop anticoagulation. Thrombophilia testing is not indicated in most family members of patients with VTE, as appropriate decisions regarding use of prophylaxis in high-risk situations and choice of contraceptive methods can be made on the basis of family history alone. Testing may be considered in female family members of patients with VTE and a known hereditary thrombophilia if results will influence choices regarding estrogen use or prophylaxis in the context of pregnancy. Table 3 summarizes these guidance statements.

**Table 3:** Summary of guidance statements

Question	Guidance statement
Secondary prevention following provoked VTE	
Should thrombophilia testing be performed to help determine duration of anticoagulation following provoked VTE?	Do not perform thrombophilia testing following an episode of provoked VTE. *Remark* A positive thrombophilia evaluation is not a sufficient basis to offer extended anticoagulation following an episode of provoked VTE.
Secondary prevention following unprovoked VTE	
Should thrombophilia testing be performed to help determine duration of anticoagulation following unprovoked VTE?	Do not perform thrombophilia testing in patients following an episode of unprovoked VTE. *Limits/exceptions* If a patient with unprovoked VTE and low bleeding risk is planning to stop anticoagulation, test for thrombophilia (Table 2) if test results would change this decision. *Remark* A negative thrombophilia evaluation is not a sufficient basis to stop anticoagulants following an episode of unprovoked VTE in a patient with low bleeding risk and willingness to continue therapy. *Remark* Heterozygosity for FVL or PGM does not increase the predicted risk of recurrence after unprovoked VTE to a clinically significant degree. See Chapter 3, “Guidance for the treatment of DVT and PE” for guidance on determining duration of anticoagulant therapy following unprovoked VTE.
Primary prevention in relatives of VTE patients	
Should family members of patients with VTE or hereditary thrombophilia undergo thrombophilia testing?	Do not test for thrombophilia in asymptomatic family members of patients with VTE or hereditary thrombophilia. *Remark* As a family history of VTE confers an excess risk of thrombosis, relatives should be counseled regarding use of prophylaxis in high risk situations.
Primary prevention in female relatives of VTE patients considering estrogen	
Should female relatives of patients with VTE or hereditary thrombophilia who are considering using estrogen-containing medications be tested for thrombophilia?	Do not test for thrombophilia in asymptomatic family members of patients with VTE or hereditary thrombophilia who are contemplating use of estrogen. *Limits/exceptions* If a woman contemplating estrogen use has a first-degree relative with VTE and a known hereditary thrombophilia (Table 2), test for that thrombophilia if the result would change the decision to use estrogen. *Remark* Family history of VTE in a first degree relative predicts an excess risk of thrombosis with estrogen use, even when thrombophilia testing is negative.
Primary prevention in female relatives of VTE patients who are contemplating pregnancy	
Should female relatives of patients with VTE or hereditary thrombophilia who are contemplating pregnancy be tested for thrombophilia?	Do not test for thrombophilia in asymptomatic family members of patients with VTE or hereditary thrombophilia who are contemplating pregnancy. *Limits/exceptions* If a woman contemplating pregnancy has a first-degree relative with VTE and a known hereditary thrombophilia (Table 2), test for that thrombophilia if the result would change VTE prophylaxis decisions. *Remark* Women with a personal history of unprovoked, estrogen-associated or pregnancy associated VTE already carry an indication for prophylaxis, and are unlikely to benefit from thrombophilia testing. *Remark* Women with multiple family members affected by VTE are more likely to carry a higher risk thrombophilia such as AT deficiency which may impact prophylaxis decisions. See Chapter 6, “Guidance for the treatment of obstetric-associated VTE” for regimens recommended for prophylaxis based on history and thrombophilia status.
Timing of thrombophilia assessment	
When thrombophilia testing is performed, at what point in the patient’s care should this be done?	Do not perform thrombophilia testing at the time of VTE diagnosis or during the initial 3-month course of anticoagulant therapy. When testing for thrombophilias following VTE, use either a 2-stage testing approach (see text) or perform testing after a minimum of 3 months of anticoagulant therapy has been completed, and anticoagulants have been held. *Remark* Pregnancy, sex and estrogen use reduce the levels of Protein S. Use of sex specific reference intervals, and testing prior to pregnancy or while not receiving estrogen preparations is preferred.

## References

[1] Egeberg O (1965). Inherited antithrombin deficiency causing thrombophilia. Thromb Diath Haemorrh.

[2] Griffin JH, Evatt B, Zimmerman TS, Kleiss AJ, Wideman C (1981). Deficiency of protein C in congenital thrombotic disease. J Clin Invest.

[3] Comp PC, Esmon CT (1984). Recurrent venous thromboembolism in patients with a partial deficiency of protein S. N Engl J Med.

[4] Dahlback B, Hildebrand B (1994). Inherited resistance to activated protein C is corrected by anticoagulant cofactor activity found to be a property of factor V. Proc Natl Acad Sci USA.

[5] Poort SR, Rosendaal FR, Reitsma PH, Bertina RM (1996). A common genetic variation in the 3’-untranslated region of the prothrombin gene is associated with elevated plasma prothrombin levels and an increase in venous thrombosis. Blood.

[6] Fechtel K, Osterbur ML, Kehrer-Sawatzki H, Stenson PD, Cooper DN (2011). Delineating the hemostaseome as an aid to individualize the analysis of the hereditary basis of thrombotic and bleeding disorders. Hum Genet.

[7] Zhou S, Welsby I (2014). Is ABO blood group truly a risk factor for thrombosis and adverse outcomes?. World J Cardiol.

[8] Rodger MA, Kahn SR, Wells PS (2008). Identifying unprovoked thromboembolism patients at low risk for recurrence who can discontinue anticoagulant therapy. CMAJ.

[9] Lutsey PL, Cushman M, Heckbert SR, Tang W, Folsom AR (2011). Longer legs are associated with greater risk of incident venous thromboembolism independent of total body height. The Longitudinal Study of Thromboembolism Etiology (LITE). Thromb Haemost.

[10] Baudouy D, Moceri P, Chiche O (2015). B blood group: a strong risk factor for venous thromboembolism recurrence. Thromb Res.

[11] Heit JA (2008). The epidemiology of venous thromboembolism in the community. Arterioscler Thromb Vasc Biol.

[12] Kearon C, Akl E (2014). Duration of anticoagulant therapy for deep vein thrombosis and pulmonary embolism. Blood.

[13] Moll S (2011). Who should be tested for thrombophilia?. Genet Med.

[14] Stegnar M (2010). Thrombophilia screening–at the right time, for the right patient, with a good reason. Clin Chem Lab Med.

[15] Nicolaides A, Hull RD, Fareed J (2013). Thrombophilia. Clin App Thromb Hemost.

[16] Christiansen SC, Cannegieter SC, Koster T, Vandenbroucke JP, Rosendaal FR (2005). Thrombophilia, clinical factors, and recurrent venous thrombotic events. JAMA.

[17] Dalen JE (2008). Should patients with venous thromboembolism be screened for thrombophilia?. Am J Med.

[18] Gohil R, Peck G, Sharma P (2009). The genetics of venous thromboembolism. A meta-analysis involving approximately 120,000 cases and 180,000 controls. Thromb Haemost.

[19] Wu O, Robertson L, Twaddle S (2006). Screening for thrombophilia in high-risk situations: systematic review and cost-effectiveness analysis. The Thrombosis: Risk and Economic Assessment of Thrombophilia Screening (Treats) Study. Health Technol Assess.

[20] Kearon C, Akl EA, Comerota AJ (2012). Antithrombotic therapy for VTE disease. Chest.

[21] Venous Thromboembolic Diseases: The Management of Venous Thromboembolic Diseases and the Role of Thrombophilia Testing. National Clinical Guideline Centre (UK). London: Royal College of Physicians 2012 23638495

[22] Baglin T, Luddington R, Brown K, Baglin C (2003). Incidence of recurrent venous thromboembolism in relation to clinical and thrombophilic risk factors: prospective cohort study. Lancet.

[23] Simone B, De Stefano V, Leoncini E (2013). Risk of venous thromboembolism associated with single and combined effects of factor V Leiden, prothrombin 20210A and methylenetethraydrofolate reductase C677T: a meta-analysis involving over 11,000 cases and 21,000 controls. Eur J Epidemiol.

[24] American College of Obstetricians Gynecologists Women’s Health Care Physicians (2013). ACOG Practice Bulletin No. 138: inherited thrombophilias in pregnancy. Obstet Gynecol.

[25] Baglin T, Gray E, Greaves M (2010). Clinical guidelines for testing for heritable thrombophilia. Br J Haematol.

[26] Jennings I, Kitchen S, Woods TA, Preston FE (2005). Multilaboratory testing in thrombophilia through the United Kingdom National External Quality Assessment Scheme (Blood Coagulation) Quality Assurance Program. Semin Thromb Hemost.

[27] Segal JB, Brotman DJ, Necochea AJ (2009). Predictive value of factor V Leiden and prothrombin G20210A in adults with venous thromboembolism and in family members of those with a mutation: a systematic review. JAMA.

[28] Hellmann EA, Leslie ND, Moll S (2003). Knowledge and educational needs of individuals with the factor V Leiden mutation. J Thromb Haemost.

[29] Cohn DM, Vansenne F, Kaptein AA, De Borgie CA, Middeldorp S (2008). The psychological impact of testing for thrombophilia: a systematic review. J Thromb Haemost.

[30] Bank I, Scavenius MP, Buller HR, Middeldorp S (2004). Social aspects of genetic testing for factor V Leiden mutation in healthy individuals and their importance for daily practice. Thromb Res.

[31] Moll S (2015). Thrombophilia: clinical–practical aspects. J Thromb Thrombolysis.

[32] Mahmoodi BK, Brouwer JL, Ten Kate MK (2010). A prospective cohort study on the absolute risks of venous thromboembolism and predictive value of screening asymptomatic relatives of patients with hereditary deficiencies of protein S, protein C or antithrombin. J Thromb Haemost.

[33] Reitter-Pfoertner S, Waldhoer T, Mayerhofer M (2013). The influence of thrombophilia on the long-term survival of patients with a history of venous thromboembolism. Thromb Haemost.

[34] Rabinovich A, Cohen JM, Prandoni P, Kahn SR (2014). Association between thrombophilia and the post-thrombotic syndrome: a systematic review and meta-analysis. J Thromb Haemost.

[35] Coppens M, Reijnders JH, Middeldorp S, Doggen CJ, Rosendaal FR (2008). Testing for inherited thrombophilia does not reduce the recurrence of venous thrombosis. J Thromb Haemost.

[36] Hicks LK, Bering H, Carson KR (2013). The ASH Choosing Wisely(R)campaign: five hematologic tests and treatments to question. Hematology.

[37] Choosing Wisely. Society for Vascular Medicine. (2014) Five Things Physicians and Pateints Should Question. http://www.vascularmed.org/choosing/SVMCWList.pdf. Accessed 20 Nov 2014

[38] Pernod G, Biron-Andreani C, Morange PE (2009). Recommendations on testing for thrombophilia in venous thromboembolic disease: a French consensus guideline. J Mal Vasc.

[39] Evaluation of Genomic Applications in Practice and Prevention Working Group (2011). Recommendations from the EGAPP Working Group: routine testing for Factor V Leiden (R506Q) and prothrombin (20210G > A) mutations in adults with a history of idiopathic venous thromboembolism and their adult family members. Gen Med.

[40] Boutitie F, Pinede L, Schulman S (2011). Influence of preceding length of anticoagulant treatment and initial presentation of venous thromboembolism on risk of recurrence after stopping treatment: analysis of individual participants’ data from seven trials. BMJ.

[41] Eichinger S, Minar E, Hirschl M (1999). The risk of early recurrent venous thromboembolism after oral anticoagulant therapy in patients with the G20210A transition in the prothrombin gene. Thromb Haemost.

[42] Eichinger S, Weltermann A, Mannhalter C (2002). The risk of recurrent venous thromboembolism in heterozygous carriers of factor V Leiden and a first spontaneous venous thromboembolism. Arch Intern Med.

[43] Couturaud F, Leroyer C, Tromeur C (2014). Factors that predict thrombosis in relatives of patients with venous thromboembolism. Blood.

[44] Tosetto A, Iorio A, Marcucci M (2012). Predicting disease recurrence in patients with previous unprovoked venous thromboembolism: a proposed prediction score (DASH). J Thromb Haemost.

[45] Eichinger S, Heinze G, Jandeck LM, Kyrle PA (2010). Risk assessment of recurrence in patients with unprovoked deep vein thrombosis or pulmonary embolism: the Vienna prediction model. Circulation.

[46] Kearon C, Spencer FA, O’Keeffe D (2015). d-Dimer testing to select patients with a first unprovoked venous thromboembolism who can stop anticoagulant therapy: a cohort study. Ann Intern Med.

[47] Donadini MP, Ageno W, Antonucci E (2014). Prognostic significance of residual venous obstruction in patients with treated unprovoked deep vein thrombosis. A patient-level meta-analysis. Thromb Haemost.

[48] Baldwin M, Moore H, Rudarakanchana N, Gohel M, Davies A (2013). Post-thrombotic syndrome: a clinical review. J Thromb Haemost.

[49] Grody WW, Griffin JH, Taylor AK, Korf BR, Heit JA, Group AFVLW (2001). American College of Medical Genetics consensus statement on factor V Leiden mutation testing. Gen Med.

[50] Merriman L, Greaves M (2006). Testing for thrombophilia: an evidence-based approach. Postgrad Med J.

[51] Foy P, Moll S (2009). Thrombophilia: 2009 update. Curr Treat Options Cardiovasc Med.

[52] Lijfering WM, Brouwer JL, Veeger NJ (2009). Selective testing for thrombophilia in patients with first venous thrombosis: results from a retrospective family cohort study on absolute thrombotic risk for currently known thrombophilic defects in 2479 relatives. Blood.

[53] Bezemer ID, van der Meer FJ, Eikenboom JC, Rosendaal FR, Doggen CJ (2009). The value of family history as a risk indicator for venous thrombosis. Arch Intern Med.

[54] Zoller B, Ohlsson H, Sundquist J, Sundquist K (2013). Familial risk of venous thromboembolism in first-, second- and third-degree relatives: a nationwide family study in Sweden. Thromb Haemost.

[55] Sorensen HT, Riis AH, Diaz LJ, Andersen EW, Baron JA, Andersen PK (2011). Familial risk of venous thromboembolism: a nationwide cohort study. J Thromb Haemost.

[56] Wu O, Robertson L, Twaddle S (2005). Screening for thrombophilia in high-risk situations: a meta-analysis and cost-effectiveness analysis. Br J Haematol.

[57] Creinin MD, Lisman R, Strickler RC (1999). Screening for factor V Leiden mutation before prescribing combination oral contraceptives. Fertil Steril.

[58] Middeldorp S (2011). Evidence-based approach to thrombophilia testing. J Thromb Thrombolysis.

[59] Rossouw JE, Anderson GL, Prentice RL (2002). Risks and benefits of estrogen plus progestin in healthy postmenopausal women: principal results From the Women’s Health Initiative randomized controlled trial. JAMA.

[60] Canonico M, Plu-Bureau G, Lowe GD, Scarabin PY (2008). Hormone replacement therapy and risk of venous thromboembolism in postmenopausal women: systematic review and meta-analysis. BMJ.

[61] James AH, Jamison MG, Brancazio LR, Myers ER (2006). Venous thromboembolism during pregnancy and the postpartum period: incidence, risk factors, and mortality. Am J Obstet Gynecol.

[62] Bates SM, Greer IA, Middeldorp S, Veenstra DL, Prabulos A-M, Vandvik PO (2012). VTE, thrombophilia, antithrombotic therapy, and pregnancy. Chest.

[63] Lussana F, Dentali F, Abbate R (2009). Screening for thrombophilia and antithrombotic prophylaxis in pregnancy: guidelines of the Italian Society for Haemostasis and Thrombosis (SISET). Thromb Res.

[64] Pabinger I, Grafenhofer H, Kaider A (2005). Risk of pregnancy-associated recurrent venous thromboembolism in women with a history of venous thrombosis. J Thromb Haemost.

[65] Heit JA, Kobbervig CE, James AH, Petterson TM, Bailey KR, Melton LJ (2005). Trends in the incidence of venous thromboembolism during pregnancy or postpartum: a 30-year population-based study. Ann Intern Med.

[66] Villani M, Tiscia GL, Margaglione M (2012). Risk of obstetric and thromboembolic complications in family members of women with previous adverse obstetric outcomes carrying common inherited thombophilias. J Thromb Haemost.

[67] Rodger MA, Hague WM, Kingdom J (2014). Antepartum dalteparin versus no antepartum dalteparin for the prevention of pregnancy complications in pregnant women with thrombophilia (TIPPS): a multinational open-label randomised trial. Lancet.

[68] Bain E, Wilson A, Tooher R, Gates S, Davis LJ, Middleton P (2014). Prophylaxis for venous thromboembolic disease in pregnancy and the early postnatal period. Cochrane Database Syst Rev.

[69] Duhl AJ, Paidas MJ, Ural SH (2007). Antithrombotic therapy and pregnancy: consensus report and recommendations for prevention and treatment of venous thromboembolism and adverse pregnancy outcomes. Am J Obstet Gynecol.

[70] Lockwood C, Wendel G, Committee on Practice Bulletins Obstetrics (2011). Practice bulletin no. 124: inherited thrombophilias in pregnancy. Obstet Gynecol.

[71] McLintock C, Brighton T, Chunilal S (2012). Recommendations for the prevention of pregnancy-associated venous thromboembolism. Aust N Z J Obstet Gynaecol.

[72] Adcock DM, Gosselin R (2015). Direct oral anticoagulants (DOACs) in the laboratory: 2015 review. Thromb Res.

[73] Pintao MC, Ribeiro DD, Bezemer ID (2013). Protein S levels and the risk of venous thrombosis: results from the MEGA case-control study. Blood.

[74] Cushman M (2014). Thrombophilia testing in women with venous thrombosis: the 4 P’s approach. Clin Chem.

[75] Lindhoff-Last E, Luxembourg B (2008). Evidence-based indications for thrombophilia screening. Vasa.

[76] Cohen W, Castelli C, Suchon P (2014). Risk assessment of venous thrombosis in families with known hereditary thrombophilia: the MARseilles-NImes prediction model. J Thromb Haemost.

[77] Davis SM, Branch DW (2010). Thromboprophylaxis in pregnancy: who and how?. Obstet Gynecol Clin North Am.

[78] Culwell KR, Curtis KM, del Carmen Cravioto M (2009). Safety of contraceptive method use among women with systemic lupus erythematosus: a systematic review. Obstet Gynecol.

[79] Wu O, Robertson L, Langhorne P (2005). Oral contraceptives, hormone replacement therapy, thrombophilias and risk of venous thromboembolism: a systematic review. The thrombosis: risk and Economic Assessment of Thrombophilia Screening (TREATS) Study. Thromb Haemost.

